# The emerging novel avian leukosis virus with mutations in the *pol* gene shows competitive replication advantages both in vivo and in vitro

**DOI:** 10.1038/s41426-018-0111-4

**Published:** 2018-06-26

**Authors:** Qi Su, Yang Li, Zhizhong Cui, Shuang Chang, Peng Zhao

**Affiliations:** 1 College of Veterinary Medicine, Shandong Agricultural University, 61 Daizong Road, 271018 Tai’an, Shandong China; 2 Shandong Provincial Key Laboratory of Animal Biotechnology and Disease Control and Prevention, 61 Daizong Road, 271018 Tai’an, Shandong China; 3 Shandong Provincial Engineering Technology Research Center of Animal Disease Control and Prevention, 271018 Tai’an, Shandong China; 4 China Animal Health and Epidemiology Center, 266032 Qingdao, Shandong China

## Abstract

The avian leukosis virus subgroup K (ALV-K), a novel subgroup in Chinese indigenous chicken breeds, has been difficult to isolate in the past due to its poor replication ability. However, according to the latest monitoring data, the replication ability and isolation rate of ALV-K have clearly increased, and new strains with mutations in the *pol* gene have also been found. To determine the effects of such mutations on the biological characteristics of ALV-K, a pair of infectious clones were constructed and rescued. The first virus was an ALV-K Chinese isolate with mutations in its *pol* gene, named rSDAUAK-11. The second virus was a recuperative rSDAUAK-11 from which mutations in the *pol* gene were recovered according to the corresponding region of the ALV-K prototype virus JS11C1, named rRSDAUAK-11. In addition, two quantitative real-time polymerase chain reaction assays were developed to specifically detect these virus strains. Using such methods, we observed a marked improvement of the reverse transcriptase activity, replication ability and vertical transmission ability of rSDAUAK-11, which also revealed a formidable competitive advantage in mixed infection with rRSDAUAK-11 and corresponded to the differences between the wild strains SDAUAK-11 and JS11C1. Accordingly, our findings not only show that mutations in the *pol* gene are an important molecular mechanism contributing to corresponding changes in the biological characteristics of the newest ALV-K but also emphasize the potential future eradication of ALV.

## Introduction

Avian leukosis is a vertically transmitted disease caused by the avian leukosis virus (ALV), which can cause serious immunosuppression, growth retardation and tumor-induced mortality. ALV infections have been reported worldwide and have caused severe economic losses to the poultry industry^1,2^. Among different Chinese chicken flocks, positive rates of ALV are universally high^3–15^, and thus, the government has launched an ALV monitoring and eradication program and demonstrated some achievements.

ALVs are divided into 11 subgroups named A–K. Viruses of subgroups A, B, and J are the most common exogenous ALVs that infect chickens in field flocks, whereas subgroups C and D are rarely reported in the field^16,17^. Subgroup E is an endogenous virus, which has low or no pathogenicity to chickens^18^. The past subgroup classification was based on the antigenicity of ALVs isolated from breeds only raised in the Euro-American region, without consideration of other countries. Nevertheless, a novel ALV subgroup was discovered from the National Chicken Genetic Resources (NCGR, Jiangsu, China)^19^. Subsequently, similar ALVs were also isolated from other Chinese indigenous chicken flocks^9,11,12,15^. It is worth noting that all these flocks were completely confined and never in contact with the outside. Thus, it is speculated that such an ALV was a specific long-standing subgroup in indigenous chicken breeds of China, namely, ALV subgroup K (ALV-K)^9,11^. The replication ability, pathogenicity and oncogenicity of ALV-K were found to be relatively low in a previous study^12^, which is prone to be ignored in the initial isolation and identification process, especially with the discovery of ALV-J in mixed infections.

However, during the most recent virus monitoring and isolation process for the NCGR in 2015, we not only detected an increase in the isolation rate of ALV-K^11^ but also found that the replication ability of the newly isolated ALV-K was clearly enhanced compared with the previous assessment. Considering that the polymerase encoded by the *pol* gene is closely related to viral replication, we speculated that some corresponding mutations therein might alter the reverse transcriptase activity and affect the replication ability.

## Results

### The mutations in the *pol* gene of the newly isolated ALV-K

**Fig. 1:**
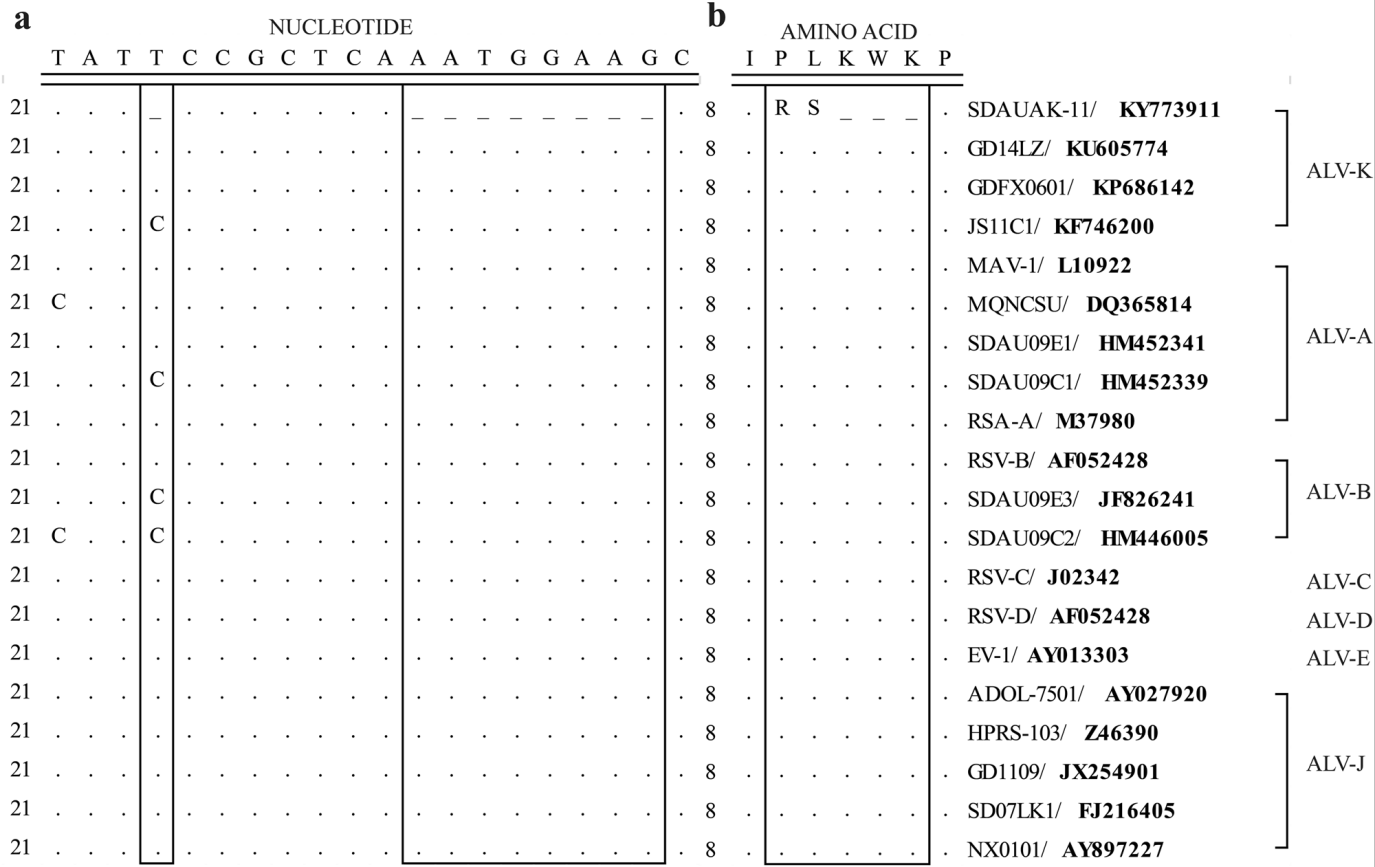
Genetic sequence comparison of the *pol* gene of SDAUAK-11 and reference ALV strains. **a** Nucleotide sequence comparison. **b** Amino acid sequence comparison. Boxes indicate the genetic difference between the SDAUAK-11 and reference strains

To determine the molecular basis of the enhanced replication ability of the newly isolated ALV-K, the *pol* gene sequences of the SDAUAK-11 and corresponding reference strains from different subgroups were compared to observe the genetic differences among them. The results showed that, compared with the prototype ALV-K strain JS11C1 and other reference strains, SDAUAK-11 has a single nucleotide deletion at position 24 and an 8-nucleotide deletion at positions 32–39 of the *pol* gene (Fig. 1a), which caused the replacement of the corresponding amino acids proline-leucine-lysine-tryptophan-lysine (P-L-K-W-K) with arginine-serine (R-S) (Fig. 1b).

### Virus rescue and identification

Two viruses, rSDAUAK-11 (with mutations in the *pol* gene) and rRSDAUAK-11 (without the mutations) were rescued using reverse genetic manipulation to determine the effects of such mutations on the biological characteristics of the novel ALV-K. The supernatant of transfected DF-1 cells was determined to be ALV-positive by ELISA analysis. The rescued virus could be passed in DF-1 cells for more than 10 generations, and sequencing of the rescued viruses, which maintained high genome stability, also demonstrated that the only difference (R-S to P-L-K-W-K) appeared in the *pol* gene between rSDAUAK-11 and rRSDAUAK-11.

### Mutations in the *pol* gene significantly enhanced reverse transcriptase activity and replication ability both in vitro and in vivo

**Fig. 2:**
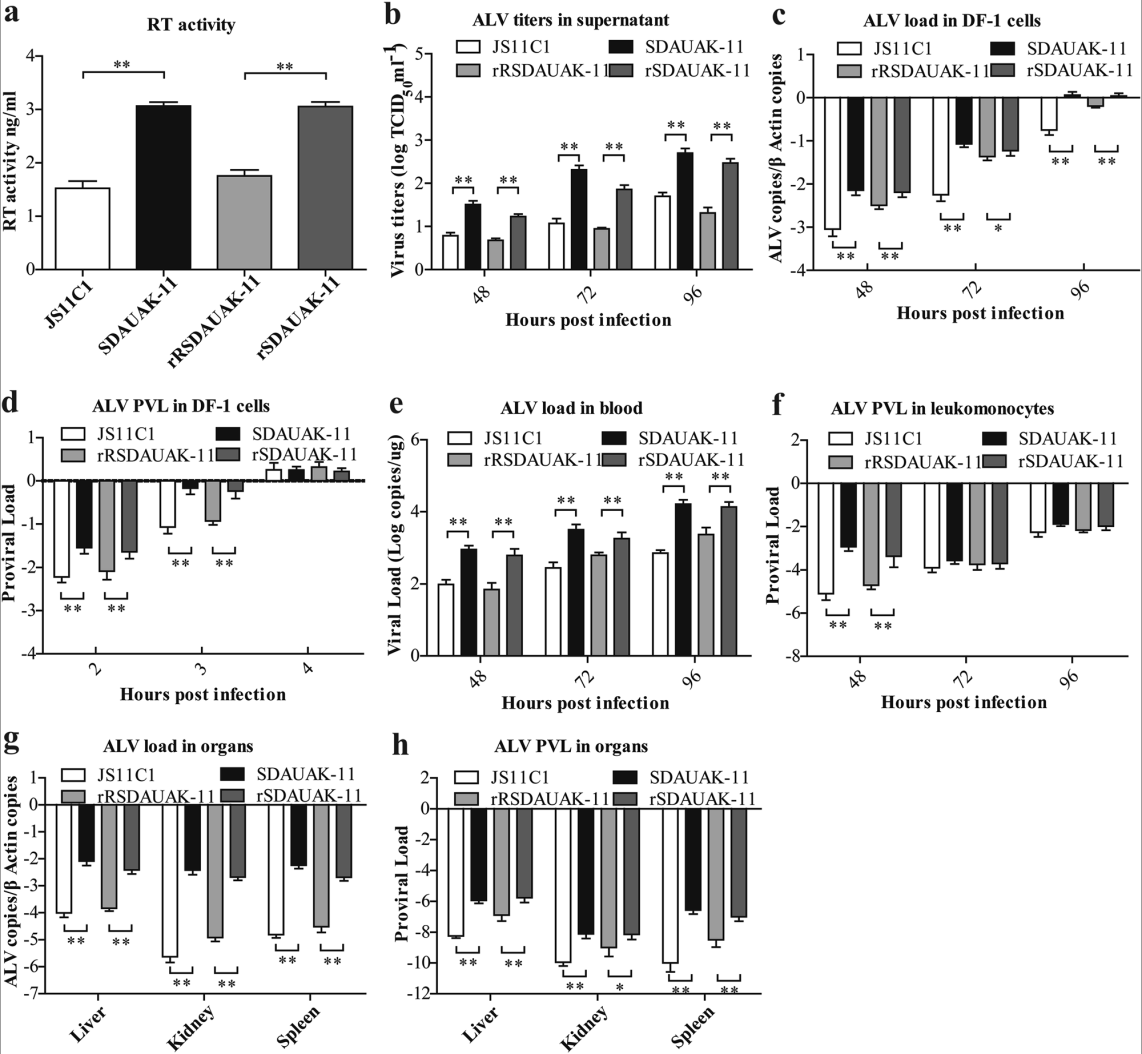
Effects of the mutations in the *pol* gene on reverse transcriptase activity and replication ability both in vitro and in vivo. **a** The results of the reverse transcriptase activity (RT) assays performed with equivalent virus particle amounts (five million copies) isolated from the cell culture supernatants of DF-1 cells infected or transfected with SDAUAK-11 (wild strain), JS11C1 (wild strain), rSDAUAK-11 and rRSDAUAK-11 are shown. **b** Virus titers for these viruses in DF-1 cell culture supernatant at 48, 72, and 96 HPI and viral titers harvested at different intervals were calculated and expressed as TCID_50_ per milliliter using Reed-Muench methods. **c** Viral loads for these viruses at 48, 72 and 96 HPI were determined by the presence of ALV in DF-1 cells (10^6^ cells) treated with different virus strains (five million copies) using QRT-PCR methods, and ALV viral load levels were normalized to *beta-actin*. **d** Proviral loads (PVLs) for those viruses at 2, 3, and 4 HPI were determined by the presence of ALV-cDNA in DF-1 cells (10^6^ cells) treated with different virus strains (five million copies) using QRT-PCR, and ALV PVL levels were normalized to *HMG14b*. **e** Viral loads for these viruses at 48, 72 and 96 HPI were determined by the presence of ALV in the blood of SPF chicks treated with different virus strains (five million copies) using QRT-PCR, and the viral RNA concentration (log_10_) was normalized per 1 µg of total RNA. **f** PVLs for those viruses at 48, 72 and 96 HPI were determined by the presence of ALV-cDNA in the leukomonocytes of SPF chicks treated with different virus strains (five million copies) using QRT-PCR methods, and ALV proviral load levels were normalized to *HMG14b*. **g** Viral loads for these viruses at 48, 72 and 96 HPI were determined by the presence of ALV in the liver, kidney and spleen of SPF chicks treated with different virus strains (five million copies) using QRT-PCR, and ALV viral load levels were normalized to *beta-actin*. **h** PVLs for these viruses at 48, 72 and 96 HPI were determined by the presence of ALV-cDNA in the liver, kidney and spleen of SPF chicks treated with different virus strains (five million copies) using QRT-PCR methods, and ALV PVL levels were normalized to *HMG14b*. The standard deviations from independent experiments are shown. *P* value (*P < 0.05, **P < 0.01) determined by Duncan’s multiple-range test is shown

To assess the effects of such mutations on reverse transcriptase (RT) activity in vitro, commensurable SDAUAK-11, JS11C1, rSDAUAK-11 and rRSDAUAK-11 with the same virion number were evaluated for RT activity using a commercial kit. The results showed that the RT activity of rSDAUAK-11 was significantly higher than that of rRSDAUAK-11 (approximately two-fold, *P* < 0.01), which was similar to the difference between the wild strains SDAUAK-11 and JS11C1 (Fig. 2a). These results illustrated that mutations in the *pol* gene are critical factor for the improvement of RT activity.

In addition, we also analyzed the dynamic state of virus titers in the cell culture supernatant at 48, 72, and 96 h post-infection (HPI) using the Reed-Muench method to demonstrate the effects of such mutations on viral shedding. The results showed that, consistent with the difference between the wild strains SDAUAK-11 and JS11C1, the viral titer of rSDAUAK-11 was clearly higher than that of rRSDAUAK-11 at all times (*P* < 0.01, Fig. 2b), which showed that SDAUAK-11 possessed high virus shedding with the help of mutations in the *pol* gene.

In contrast, quantitative real-time PCR (QRT-PCR) analysis revealed that the viral loads of SDAUAK-11 in DF-1 cells were significantly higher than those of JS11C1 (*P* < 0.05), and a similar but reduced difference was also observed for the rescued viruses (Fig. 2c), which demonstrated that SDAUAK-11 was capable of higher replication ability and that mutations in the *pol* gene played an important role in such improvement. Moreover, the proviral loads (PVLs) of these viruses in DF-1 cells were also employed to show the reverse transcription ability of the newest ALV-K and its relationship with the mutations in the *pol* gene. The results showed that the PVL of SDAUAK-11 was significantly higher than that of JS11C1 at 2 or 3 HPI (*P* < 0.01), and similar results were obtained for the rescued viruses (Fig. 2d), which demonstrated that the enhanced RT activity resulting from mutations in the *pol* gene promoted the reverse transcription process of the novel ALV-K and accelerated its replication process. However, the abovementioned difference disappeared at 4 HPI (Fig. 2d), which showed that the totality of ALV-cDNA integration sites in DF-1 cells was certain, and thus, the faster reverse transcription process would help the virus occupy more sites in competition with other similar or different viruses.

To further assess the effects of such mutations in the *pol* gene on the biological activity of the newest ALV-K, an in vivo model using specific pathogen-free (SPF) chickens was also established. The same methods used in the abovementioned trials were also employed to determine the viral load in the blood or different organs and the PVL in leukomonocytes, as well as different organs. Not surprisingly, the viral load and PVL of SDAUAK-11 were all higher than that of JS11C1 in the blood, liver, kidney and spleen (*P* < 0.05), and corresponding trials using the two rescued viruses provided similar results (Fig. 2e–h).

### Mutations in the *pol* gene significantly improved the competition advantages both in vitro and in vivo

**Fig. 3:**
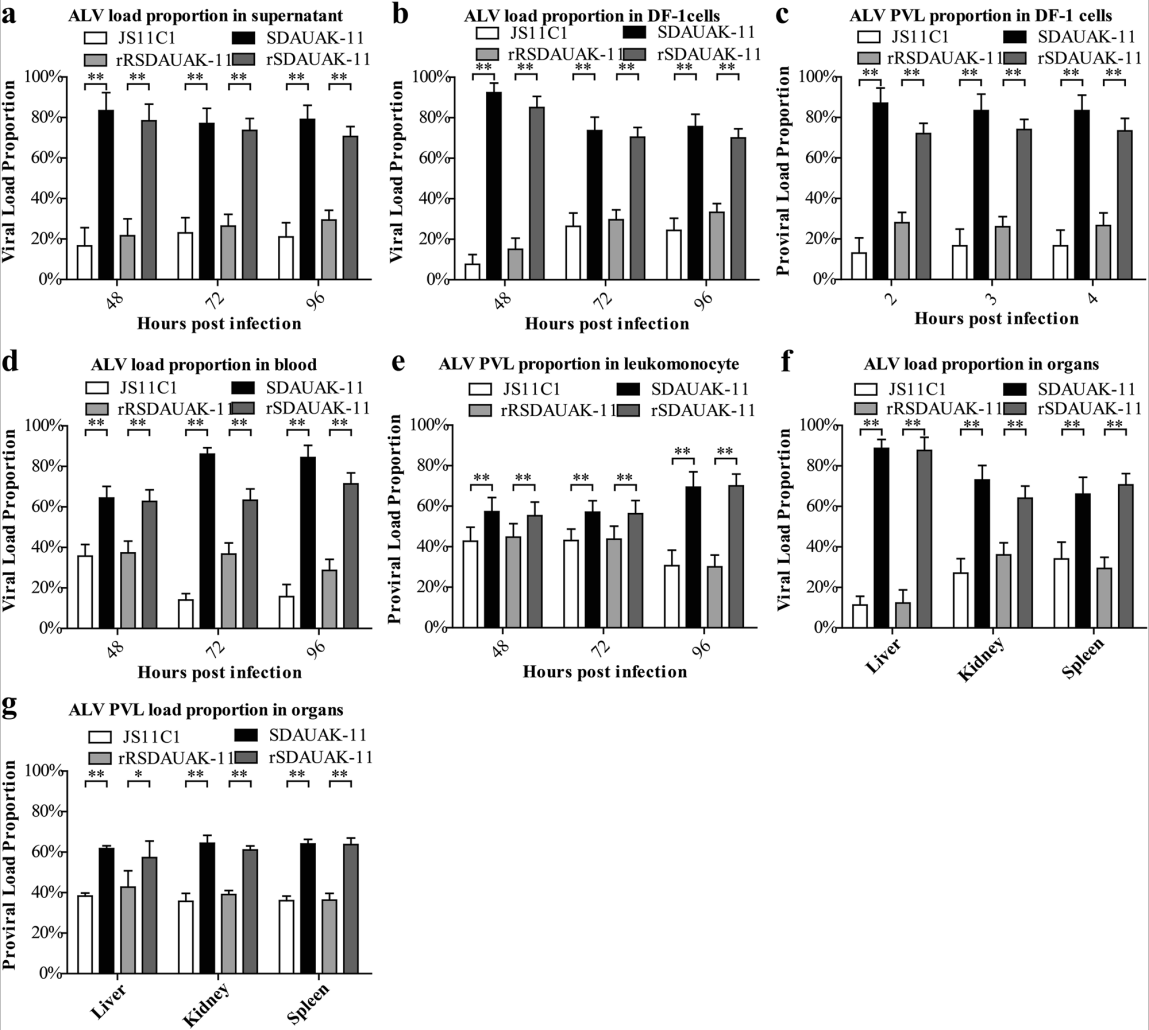
Effects of mutations in the *pol* gene on the competition advantage both in vitro and in vivo **a** Viral load proportion (VLP) for rSDAUAK-11, rRSDAUAK-11, SDAUAK-11 (wild strain), and JS11C1 (wild strain) in DF-1 cells (10^6^ cells) treated simultaneously with these viruses (five million copies and five million copies) or culture supernatant at 48, 72 and 96 HPI, and the VLP of each sample harvested at different intervals was calculated as VLP (JS11C1) = VL (JS11C1)/[VL (SDAUAK-11) + VL (JS11C1)]. Primers rR-F and rR-R were used to quantify the viral load of JS11C1, and primers r-F and r-R were used to quantify the total viral load of SDAUAK-11 and JS11C1. The same methods were used to analyze the rescued viruses and subsequent tests. **b** VLP for ALVs in DF-1 cells treated simultaneously with two wild/rescued ALVs at 48, 72, and 96 HPI. **c** PVL proportion for ALVs in DF-1 cells treated simultaneously with two wild/rescued ALVs at 48, 72, and 96 HPI. **d** VLP for ALVs in the blood of SPF chicks treated simultaneously with two wild/rescued ALVs. € PVL for ALVs in leukomonocytes of SPF chicks treated simultaneously with two wild/rescued ALVs. **f** VLP for ALVs in liver, kidney and spleen of SPF chicks treated simultaneously with two wild/rescued ALVs. **g** PVL proportion for ALVs in liver, kidney and spleen of SPF chicks treated simultaneously with two wild/rescued ALVs. The standard deviations from independent experiments are shown. *P* value (*P < 0.05, **P < 0.01) determined by Duncan’s multiple-range test is shown

To demonstrate the effects of the mutation in the *pol* gene on the competition ability of the newest ALV-K, artificial simulation mixed infection experiments were performed using wild strains SDAUAK-11 and JS11C1 and the rescued viruses rSDAUAK-11 and rRSDAUAK-11, as well as the abovementioned two QRT-PCR methods. In vitro experiments showed that the viral load proportion (VLP) of SDAUAK-11 was significantly higher than that of JS11C1 at 48, 72, and 96 HPI both in the cell culture supernatant (*P* < 0.01, Fig. 3a) and DF-1 cells (*P* < 0.01, Fig. 3b), and the PVL proportion of SDAUAK-11 was also higher than that of JS11C1 at 2, 3, and 4 HPI (*P* < 0.01, Fig. 3c); similar results were also obtained in corresponding trials using the rescued viruses (Fig. 3a–c). It is worth noting that, inconsistent with the PVL differences observed in single infection trials (Fig. 2d), the PVL proportion of SDAUAK-11/rSDAUAK-11 was higher than that of JS11C1/rRSDAUAK-11 at all times, indicating that the former always occupied a higher ALV-cDNA integration site proportion than the latter in the mixed infection experiments. In contrast, the in vivo experiments also showed similar and regular patterns. Specifically, the VLPs in the blood, liver, kidney and spleen and the PVL proportion in the leukomonocytes, liver, kidney, and spleen of SDAUAK-11/rSDAUAK-11 were significantly increased compared with JS11C1/rRSDAUAK-11 (Fig. 3d–g).

### Mutations in the *pol* gene significantly elevated the vertical transmission ability of ALV-K

Considering that the viral replication ability was closely related to virulence, it is also necessary to determine the effects of mutations in the *pol* gene on the pathogenicity of the newest ALV-K. We analyzed the pathogenicity of JS11C1, SDAUAK-11, rSDAUAK-11, and rRSDAUAK-11 and their vertical transmission ability in detail via systemic animal experiments. The results showed no obvious differences in growth inhibition, immunosuppression, or clinical symptoms caused by the viruses (data not shown) in the primary generation, but the ALV-positive rates in meconium, blood, and cloaca of group SDAUAK-11 were consistently higher than those of group JS11C1 at all times; similar results were obtained for group rSDAUAK-11 and rRSDAUAK-11 (Table 1), which demonstrated that the higher replication ability caused by mutations in the *pol* gene led to a higher ALV-positive rate. More importantly, in the offspring generation, the ALV-positive rate in meconium, blood, and cloaca of group SDAUAK-11 was also higher than that of group JS11C1, and similar results were obtained in group rSDAUAK-11 and rRSDAUAK-11, which suggested that the mutations in the *pol* gene significantly improved the vertical transmission ability of ALV-K (Table 1).

**Table 1:** ALV-positive rate of different groups both in the primary and offspring generations

Generations	Groups	ALV-positive rate (%)						
		Meconium	Blood			Cloaca		
			5 weeks	15 weeks	25 weeks	5 weeks	15 weeks	25 weeks
Primary	JS11C1	73.33	70.00	50.00	40.00	43.33	20.00	20.00
	SDAUAK-11	80.00	76.67	60.00	53.33	53.33	30.00	36.67
	rRSDAUAK-11	86.67	53.33	50.00	43.33	30.00	0.00	6.67
	rSDAUAK-11	76.67	70.00	56.67	50.00	36.67	6.67	16.67
Offspring	JS11C1	3.00	0.00	–	–	1.00	–	–
	SDAUAK-11	9.00	4.00	–	–	6.00	–	–
	rRSDAUAK-11	1.00	1.00	–	–	1.00	–	–
	rSDAUAK-11	4.00	5.00	–	–	7.00	–	–

## Discussion

According to the long-term epidemiological investigation, the most common ALVs in China are ALV-J and ALV-A^4,5,7,10,13,14,20–22^, which were also detected during the ALV eradication process in the NCGR. However, recently, a novel subgroup was found in the NCGR and named ALV-K^9^. Considering the completely confined status of the NCGR, we deemed that ALV-K was a specific long-standing subgroup in Chinese indigenous chicken breeds, which tended to be masked by ALV-J in the past due to its low replication ability and competitive edge^12^. However, along with the eradication of ALV-J, the newest ALV-K with relatively high replication ability was observed and came to our attention.

More importantly, compared with previous strains, the newest ALV-K strain isolated from the NCGR in 2015 has some arresting mutations, which are located in the *pol* gene, the most conservative gene of ALV. Specifically, a single nucleotide deletion exists at position 24 and an 8-nucleotide deletion at positions 32–39, which cause the replacement of the corresponding amino acids P-L-K-W-K with R-S. In a previous study, a mutation in the highly conserved *pol* gene blocked incorporation of the *gag-pol* polyprotein into virions, which led to replication-defective and noninfectious virions^23^. However, in the newest ALV-K in this study, mutations did not preclude the expression and translation of the *pol* gene and became a stable hereditary feature. Simultaneously, the rate of viral isolation and replication ability in DF-1 cells of ALV-K were clearly increased in many other Chinese indigenous chicken breeds^11^.

The reverse transcriptase and integrase encoded by the *pol* gene play an important role in the reverse transcription and integration process of ALV after infection^24^, which is essential for viral proliferation. Moreover, the higher order structure of a protein is determined by its primary sequence and influences protein function. For example, a mutation in the RNA-dependent RNA polymerase function domain of HEV could significantly improve its replication ability^25^. Thus, the primary sequence change by mutations in the *pol* gene must influence the higher structure and protein function of the *pol* gene-encoded proteins. To examine the effects of such mutations on the biological characteristics of ALV-K, bioinformatics analysis was used to predict the secondary and tertiary protein structure of the *pol* gene*-*encoded proteins. Specifically, compared with rRSDAUAK-11, mutations of the *pol* gene in rSDAUAK-11 induced a percentage decrease in the strand structure, as well as a percentage increase in the loop structure, altering the protein hydrophilicity and surface charge distribution by influencing the scale of the solvent accessibility sites and changing some putative protein binding regions or polynucleotide binding regions (Fig. S1). In contrast, prediction of the tertiary protein structure shows that the 9-14 amino acid positions of the reverse transcriptase both of rSDAUAK-11 and rRSDAUAK-11 are all located on the protein surface (Fig. S2). Thus, we hypothesized that such a mutation might influence *pol* protein activity and binding capacity.

To further study the effects of the mutations on RT activity and replication ability, two viruses, including rSDAUAK-11 and rRSDAUAK-11, were rescued using reverse genetic manipulation. Additionally, SDAUAK-11, JS11C1, rSDAUAK-11 and rRSDAUAK-11 were tested and compared in vitro. The results showed that the RT activity, replication ability and virus shedding were significantly higher in rSDAUAK-11 and SDAUAK-11 than in rRSDAUAK-11 and JS11C1, which indicated that such mutations significantly improved the reverse transcriptase activity and replication ability and promoted early viral proliferation and shedding. The aforementioned phenomena were also repeated and subsequently verified in vivo; specifically, the replication ability of rSDAUAK-11 was clearly higher than that of rRSDAUAK-11 in SPF chickens, similar to the difference between wild strains SDAUAK-11 and JS11C1.

Numerous mutations can occur during the replication process of a virus, especially in retroviruses, but not all mutations become heritably stable. Only those that help the virus acquire new functions or competitive advantages can survive in the grim virus competition, which is dominated by survival of the fittest. According to previous reports, a 19-bp insertion in the 5’ noncoding region could improve the replication ability of ALV-J in DF-1 cells^26^, while a 205-bp deletion in the 3’ noncoding region greatly increased the oncogenicity and lethality rates^27^, which illustrated that all preserved mutations of the ALV genome over the lengthy evolutionary process changed the biological functions and features of the virus. To verify the competitive advantage caused by mutations in the *pol* gene, two QRT-PCR assays were developed to accurately evaluate the replication trends and proportion relation of SDAUAK-11, JS11C1, rSDAUAK-11, and rRSDAUAK-11 in the mixed infections both in vitro and in vivo. As anticipated, similar to the wild strains, rSDAUAK-11 exhibited a higher viral load proportion in DF-1 cells, blood, leukomonocytes and different organs and reduced the rRSDAUAK-11 territory, providing supportive evidence for the improved rate of viral isolation of the newest ALV-K as SDAUAK-11.

In contrast, the effects of the higher replication ability caused by mutations in the *pol* gene on the pathogenicity of the newest ALV-K are also of our interest considering that viral replication ability is closely related to virulence. Although no obvious differences were observed in growth inhibition, immunosuppression, or clinical symptoms for these viruses, a systemic animal experiment showed that such mutations significantly improved the vertical transmission ability of the newest ALV-K, which might cause its further diffusion and prevalence.

In conclusion, mutations in the *pol* gene improved the reverse transcriptase activity and increased the replication rate of SDAUAK-11 both in vitro and in vivo, which conferred a competitive edge, higher virus shedding and vertical transmission ability to the newest ALV-K. This study demonstrated the effects of such mutations on the biological functions and features of the newest ALV-K and helped us further understand the evolution and development of ALV. Moreover, we believe that ALV-K with similar mutations might have appeared in different indigenous chicken breeds. Thus, this study also serves a reminder to strengthen the monitoring of ALV-K in China to avoid the further spread of ALV-K with higher replication and vertical transmission abilities similar to SDAUAK-11 and evade potential associated risks, especially in the current critical stage of the ALV eradication project in China.

## Materials and methods

### Virus background

JS11C1 was isolated from NCGR in 2012 (GenBank accession number: KF746200), and SDAUAK-11 was recently isolated from the same Chinese indigenous chicken flock in 2015 (GenBank accession number: KY773911), both of which belong to the novel ALV-K^27,28^, but we noted that the replication ability of SDAUAK-11 was higher than that of JS11C1 during the primary virus isolation process. All virus stocks were stored at −80 °C in our laboratory until further analysis.

### Comparison of the *pol* gene of SDAUAK-11 and other reference strains

The *pol* gene sequences of SDAUAK-11 and other reference strains of different subgroups were obtained from GenBank. The strain information is listed in Table S1. Multiple sequence alignment of these sequences was performed using the Clustal W (BioEdit version 7.0) program.

### Construction of rSDAUAK-11 and rRSDAUAK-11

**Fig. 4:**
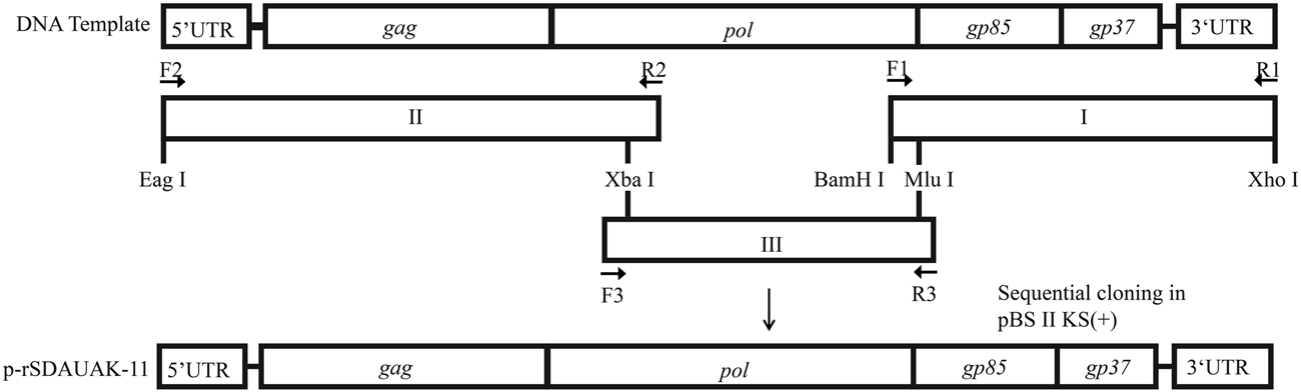
Schematic diagrams showing the construction of p-rSDAUAK-11 and p-rRSDAUAK-11. F1/R1, F2/R2, and F3/R3 were primers used for amplification of the whole genome of SDAUAK-11. Xba I and Mlu I were restriction enzyme sequences located in the genome itself, while Eag I, BamH I, and Xho I were incorporated into the amplicons. With the exception of Mlu I, the other four restriction enzymes can be found in pBlueScript II KS. Amplicons I, II, and III were inserted into pBlueScript II KS in order using restriction enzyme digestion. Amplicon I was first inserted into pBlueScript II KS via BamH I and Xho I; amplicon II was then inserted via Eag I and Xba I; and finally, amplicon III was inserted into the genome itself via Xba I and Mlu I

The construction strategy for the two full-length cDNA clones is illustrated in Fig. 4. Proviral DNA was extracted from SDAUAK-11-infected, as well as JS11C1-infected DF-1 cells for use as PCR templates. The SDAUAK-11 genome was cleaved into three fragments using the Xba I and Mlu I restriction enzymes and amplified separately. The primers used to amplify fragments I, II. and III are shown in Table 2. The Xba I and Mlu I restriction enzyme sites present in the genome sequence itself and the Eag I, Bam HI, Xho I restriction enzyme sites introduced by PCR primers were used. The amplicons were gel-purified and subcloned into the PMD-19T vectors (TaKaRa, Dalian, China) and then inserted into the pBlueScript II KS (+) plasmid (TaKaRa) in the correct orientation by digestion with appropriate restriction enzymes (Takara). The recombinant plasmid was named p-rSDAUAK-11. In addition, to recover the nucleotide deletions in the *pol* gene, primers Fr2, Rr2; F2, Rr1; and Fr1, R2 were used for amplification of fragment 2714-3119 of JS11C1 (containing the deleted nucleotides in the same positions and no unrelated amino acid sequence differences between SDAUAK-11 and JS11C1, Fig. S3), fragment 1-2728 of SDAUAK-11 and fragment 3048-3647 of SDAUAK-11, respectively. All these small fragments were combined into a new fragment II, designated rII, through gene splicing by overlap extension PCR (SOEPCR). Fragment rII was inserted into the pBlueScript II KS (+) plasmid in the correct orientation by digestion with appropriate restriction enzymes with fragments I and III, and the recombinant plasmid was named p-rRSDAUAK-11. Additionally, all amplicons were sequenced to verify the accuracy of the PCR-amplified regions, and p-rRSDAUAK-11 was sequenced to verify the recovery of the abovementioned mutations and exclude the existence of an unrelated mutation.

**Table 2:** Primers used in this study

Primer^a^	Sequence	Length
F1	AAA***GGATCC***ACGCGTAATGTAGCCTTACACAATAGC	1821 bp
R1	CC***CTCGAG***TCACGAGATTTTCGCATGGGAATTC	
F2	AAA***CGGCCG***TCGGAATTCCCATGCGAAAATC	3647 bp
R2	TTCCGAGAGGGTACCCAAATAAC	
F3	CTGATAAGGTTATTTGGGTACCCTCTC	2576 bp
R3	GTCGACTGAAGCCTTCTGCTTCATT	
Fr1	ATGGTCCTAGACCTCAAGGA	
Rr1	TCCAACAACTAAGTGAAGGT	
Fr2	ACTGTTCTTACTGTTGCGCTAC	390 bp
Rr2	CCTGGTTATTCACAGAG	
r-F	AACACTGGAGACCTACCGTTCTTA	207 bp
r-R	ATCTTTCTTAGTCACCTCAGCCTTT	
rR-F	CGAGTCGCTCAAATGGAAG	191 bp
rR-R	TCCGAAGGCCCAGAATAGCGAATA	
*HMG14b*-F	ACTGAAGAGACAAACCAAGAGC	212 bp
*HMG14b*-R	CCAGCTGTTTTAGACCAAAGAA TAC	
*beta*-F	TGAAGCCCAGAGCAAAAGAGGTAT	185 bp
*beta*-R	TGCTCCTCAGGGGCTACTCTC	

^a^F and R represent upstream and downstream primers, respectively. The boldface, italic parts are BamH I (F1), Xho I (R1) and Eag I (F2) restriction enzyme sites

### Viral rescue and identification

Highly purified p-rSDAUAK-11 and p-rRSDAUAK-11 plasmids were prepared using QIAGEN Plasmid Midi kits (Qiagen, Hilden, Germany) according to the manufacturer’s instructions. Each of the purified p-rSDAUAK-11 and p-rRSDAUAK-11 with 4 µg plasmids was transfected into DF-1 cells using Lipofectamine 2000 (Invitrogen, Carlsbad, CA) according to the manufacturer’s instructions. The culture supernatant of each virus stock was harvested separately at 48 h post-transfection and then blindly passed into the secondary DF-1 cells. After seven days, the secondary DF-1 cell supernatants were analyzed by an ALV group-specific antigen (p27) ELISA (IDEXX, Inc., Westbrook, MA, USA). The positive cell culture supernatants were then collected and centrifuged at 6000 r.p.m. for 5 min in an Eppendorf tube to remove floating cells and stored at −80 °C as trial samples until assayed. The rescued viruses were named rSDAUAK-11 and rRSDAUAK-11 and then continuously passed in DF-1 cells for confirmation of their proliferation stability and genome stability by whole-genome sequencing.

### QRT-PCR assay method design

The harvested rSDAUAK-11 and rRSDAUAK-11 supernatant samples produced in the DF-1 cells were used for viral genomic RNA isolation using an RNA extraction kit according to the manufacturer’s instructions (Omega, USA). The amount of RNA was quantified using a NanoDrop Spectrophotometer ND-1000 (Thermo Fisher Scientific, Waltham, MA, USA), and a 10-fold serial dilution of the genomic RNA was made for reverse transcription to cDNA as a standard template (Takara). The upstream primer (rR-F) for rRSDAUAK-11 and JS11C1 was located in the 32–39-nucleotide position of the *pol* gene, where it contains eight successive different nucleotides. Additionally, sectional nucleotides were added to the 5’ terminus to strengthen the primer stability and specificity. The downstream primer (rR-R) was selected based on the annealing temperature of the upstream primer for amplification efficiency. The QRT-PCR cycling conditions were 95 °C for 5 min, followed by 40 cycles of 95 °C for 5 s and 62 °C for 34 s. Moreover, another pair of primers (r-F, r-R) was used to simultaneously quantify rSDAUAK-11, rRSDAUAK-11, SDAUAK-11 and JS11C1, and the cycling conditions for the QRT-PCR were 95 °C for 5 min, followed by 40 cycles of 95 °C for 5 s and 60 °C for 40 s. The sequences of all primers used are shown in Table 2. Real-time PCR was carried out in a final volume of 20 µL containing 10 µL of 2 × reaction buffer, 0.5 µM forward primer, 0.5 µM reverse primer, 0.4 µL of ROX, 1 µL of template and double-distilled water to 20 µL using Q SYBR green premix according to the manufacturer’s protocol (Takara). The assays were performed on the ABI 7500 PCR machine (Thermo Fisher, USA). QRT-PCR reactions were performed in duplicate for each sample, with each sample present in technical duplicate during each run. All QRT-PCR reactions were performed with a no-template control.

### PVL quantification

PVL was measured by QRT-PCR of ALV for *pol* (primers r-F and r-R) and *HMG14b* (primers *HMG14b*-F and *HMG14b*-R) using Q SYBR green premix (Takara) according to the manufacturer’s protocol. *HMG14b* is a known single-copy gene in the chicken genome and was thus used as a housekeeping reference gene^29^. Thermal cycling conditions were 95 °C for 5 min and 40 cycles each of 95 °C for 5 s followed by 58 °C for 30 s. The reactions were also performed on an ABI 7500 PCR machine (Thermo Fisher, USA).

### Analysis of reverse transcriptase activity

The stored virus stocks of SDAUAK-11, JS11C1, rSDAUAK-11, and rRSDAUAK-11 (ALV-positive cell culture supernatant from infected or transfected DF-1 cells) were analyzed using the above-established QRT-PCR assays (primers r-F and r-R), first to determine the viral concentration and corresponding dilution factors. Next, these stocks at the same virion number (five million copies) were analyzed using a colorimetric RT assay (Roche Applied Science, Indianapolis, IN) to quantitate the RT activity according to the manufacturer’s protocol.

### Analysis of replication ability in vitro

Eight 6-well cell culture plates with a monolayer of DF-1 cells were prepared before infection and designated A1, A2, B1, B2, C1, C2, D1, and D2. Five million copies of SDAUAK-11 were inoculated into each A1 and A2 well, five million copies of JS11C1 into B1 and B2, five million copies of rSDAUAK-11 into C1 and C2, and five million copies of rRSDAUAK-11 into D1 and D2. The supernatants from each well of A1, B1, C1, and D1 were collected at 48, 72, and 96 HPI to determine the virus titer as the TCID_50_ mL^-1^ using the Reed-Muench method^30^. The cells from each well of A1, B1, C1, and D1 were collected at 48, 72, and 96 HPI to determine the viral load by RNA extraction using an RNA extraction kit (Omega) following the above-established QRT-PCR assays (primers r-F and r-R), and viral load levels were normalized to *beta-actin* (primers *beta*-F and *beta*-R). Additionally, the cells from each well of A2, B2, C2, and D2 were collected at 2, 3, and 4 HPI to determine the PVL by DNA extraction using a DNA extraction kit (Omega) following the abovementioned PVL quantification methods. Each experiment was repeated independently three times, and each sample was tested independently three times.

### Analysis of replication ability in vivo

Forty one-day-old specific pathogen-free (SPF) chicks (SPAFAS poultry company, Jinan, China) were randomly divided into four groups of 10 chicks per group and separately bred in shielded cages with positive filtered air. Chicks in each group were intravenously inoculated with equal amounts of SDAUAK-11, JS11C1, rSDAUAK-11, or rRSDAUKA-11 (five million copies). Blood with anti-coagulant was collected from each chick for RNA extraction and viral load quantification at 48, 72 and 96 HPI, and the viral RNA concentrations were normalized per 1 µg of total RNA. Leukomonocytes were also extracted from the blood using lymphocyte separation medium (Solarbio, USA) for DNA extraction, followed by PVL quantification. All chicks were dissected at 7 days of age to collect samples of liver, thymus, bursa of Fabricius, spleen, lung, kidney, intestine and other organs for RNA extraction and viral load quantification, while the viral loads were normalized to *beta-actin*, and DNA extraction followed PVL quantification. Each sample was tested independently three times.

### Analysis of the competition advantage in vitro

Four 6-well cell culture plates with a monolayer of DF-1 cells were prepared before infection and designated E1, E2, F1, and F2. Five million copies of SDAUAK-11 and an equal amount of JS11C1 were inoculated into each E1 and E2 well, while five million copies of rSDAUAK-11 and an equal amount of rRSDAUAK-11 were inoculated into each F1 and F2 well. The supernatants and cells from each well of E1 and F1 were collected at 48, 72 and 96 HPI to extract RNA as template, and the cells from each well of E2 and F2 were collected at 2, 3, and 4 HPI to extract DNA as template. Each template was analyzed twice using the above-established two QRT-PCR methods, and the results for each sample were calculated as VLP (JS11C1) = VL (JS11C1)/[VL (SDAUAK-11) + VL (JS11C1)] or VLP (rRSDAUAK-11) = VL (rRSDAUAK-11)/[VL (rSDAUAK-11) + VL (rRSDAUAK-11)]. Each sample was tested independently three times.

### Analysis of the competition advantage ability in vivo

Twenty one-day-old SPF chicks (SPAFAS) were randomly divided into two groups with 10 chicks per group and separately bred in shielded cages with positive filtered air. Chicks in each group were intravenously inoculated with five million copies of SDAUAK-11 and an equal amount of JS11C1 or five million copies of rSDAUAK-11 and an equal amount of rRSDAUAK-11. Blood with anti-coagulant was collected at 48, 72, and 96 HPI to extract RNA as template, while leukomonocytes were also collected at the same intervals to extract DNA as template. All chicks were dissected at 7 days of age to collect samples of liver, thymus, bursa of Fabricius, spleen, lung, kidney, intestine and other organs for RNA extraction, as well as DNA extraction as a template. Each template was analyzed twice using the above-established two QRT-PCR methods, and the results for each sample were calculated as described above. Each sample was tested independently three times.

### Analysis of the pathogenicity of the newest ALV-K

One hundred and twenty SPF embryonated eggs (SPAFAS) were randomly divided into four groups, with thirty embryonated eggs per group, and hatched in an incubator until 5 embryonic days of age. Each embryonated egg was then inoculated with equal amounts of SDAUAK-11, JS11C1, rSDAUAK-11, and rRSDAUAK-11 (five million copies) in the yolk sac, respectively. After hatching, meconium samples were collected from each chick to detect ALV using an ALV group-specific antigen (p27) ELISA (IDEXX), while blood and cloaca cotton swabs were collected from each chick at 5, 15, and 25 weeks of age to detect ALV using the above-established QRT-PCR (primers r-F and r-R). On the other hand, since 20 weeks of age, artificial inseminations were performed in each group to observe the vertical transmission ability of the viruses, and one hundred offspring embryonated eggs from each group were collected and hatched in the same incubator. After hatching, all chicks were examined using the same methods to detect ALV. Moreover, detailed data for growth inhibition, immunosuppression and clinical symptoms were also collected using conventional methods.

### Statistical analysis

Statistical analysis was performed using the SPSS statistical software package for Windows version 17.0 (SPSS Inc. Chicago, Illinois, USA). *P* < 0.05 was considered statistically significant based on Duncan’s multiple-range test.

### Ethics statement

The animal care and use protocol was approved by the Shandong Agricultural University Animal care and use Committee (SDAUA-2016-002). All experimental animals in this study were cared for and maintained throughout the experiments strictly following the ethics and biosecurity guidelines approved by the Institutional Animal Care and Use Committee of Shandong Agricultural University.

## Electronic supplementary material

Table S1 Avian leukosis virus strains used in this study

Fig. S1 Secondary protein structure prediction using the online software PredictProtein (https://www.predictprotein.org/) (a) Secondary protein structure element distribution map of rSDAUAK-11 (b) sec

Fig. S2 Tertiary protein structure prediction using the online software SWISS-MODEL (https://www.swissmodel.expasy.org/) (a) rSDAUAK-11 (b) rRSDAUAK-11; the 1-13 (rSDAUAK-11: TVALHLAIRSPDH) and 1-16

Fig. S3 Amino acid sequence comparison of fragment 2714-3119 between JS11C1 and SDAUAK-11, and the difference was marked with black rectangle
