# Supplementary material for: The emerging novel avian leukosis virus with mutations in the pol gene shows competitive replication advantages both in vivo and in vitro
Source: Emerg Microbes Infect. 2018 Jun 26;7:117. doi: 10.1038/s41426-018-0111-4 (PMC6018675; doi:10.1038/s41426-018-0111-4)
Supplement: Supplementary file 1 — Table S1 Avian leukosis virus strains used in this study [file 41426_2018_111_MOESM1_ESM.docx]

**Table S1 Avian leucosis virus strains used in this study**

| No. | Sub  group | Strain | Origin | Accession no. | No. | Sub  group | Strain | Origin | Accession no. |
| --- | --- | --- | --- | --- | --- | --- | --- | --- | --- |
| 1 | A | MQNCSU | USA | DQ365814 | 11 | E | Ev-1 | USA | AY013303 |
| 2 | A | RAV-A | France | M37980 | 12 | J | ADOL7501 | USA | AY027920 |
| 3 | A | SDAU09E1 | China | HM452341 | 13 | J | GD1109 | China | JX254901 |
| 4 | A | MAV-1 | USA | L10922 | 14 | J | HPRS-103 | UK | Z46390 |
| 5 | A | SDAU09C1 | China | HM452339 | 15 | J | NX0101 | China | AY897227 |
| 6 | B | RSV-SR-B | USA | AF052428 | 16 | J | SD07LK1 | China | FJ201640 |
| 7 | B | SDAU09E3 | China | JF826241 | 17 | K | JS11C1 | China | KF746200 |
| 8 | B | SDAU09C2 | China | HM446005 | 18 | K | GD14LZ | China | KU605754 |
| 9 | C | RSV-PragueC | USA | J02342 | 19 | K | GDFX0601 | China | KP686142 |
| 10 | D | RSV-SR-D | USA | D10652 |  |  |  |  |  |
