# Supplementary figures and images for: The emerging novel avian leukosis virus with mutations in the pol gene shows competitive replication advantages both in vivo and in vitro
Source: Emerg Microbes Infect. 2018 Jun 26;7:117. doi: 10.1038/s41426-018-0111-4 (PMC6018675; doi:10.1038/s41426-018-0111-4)

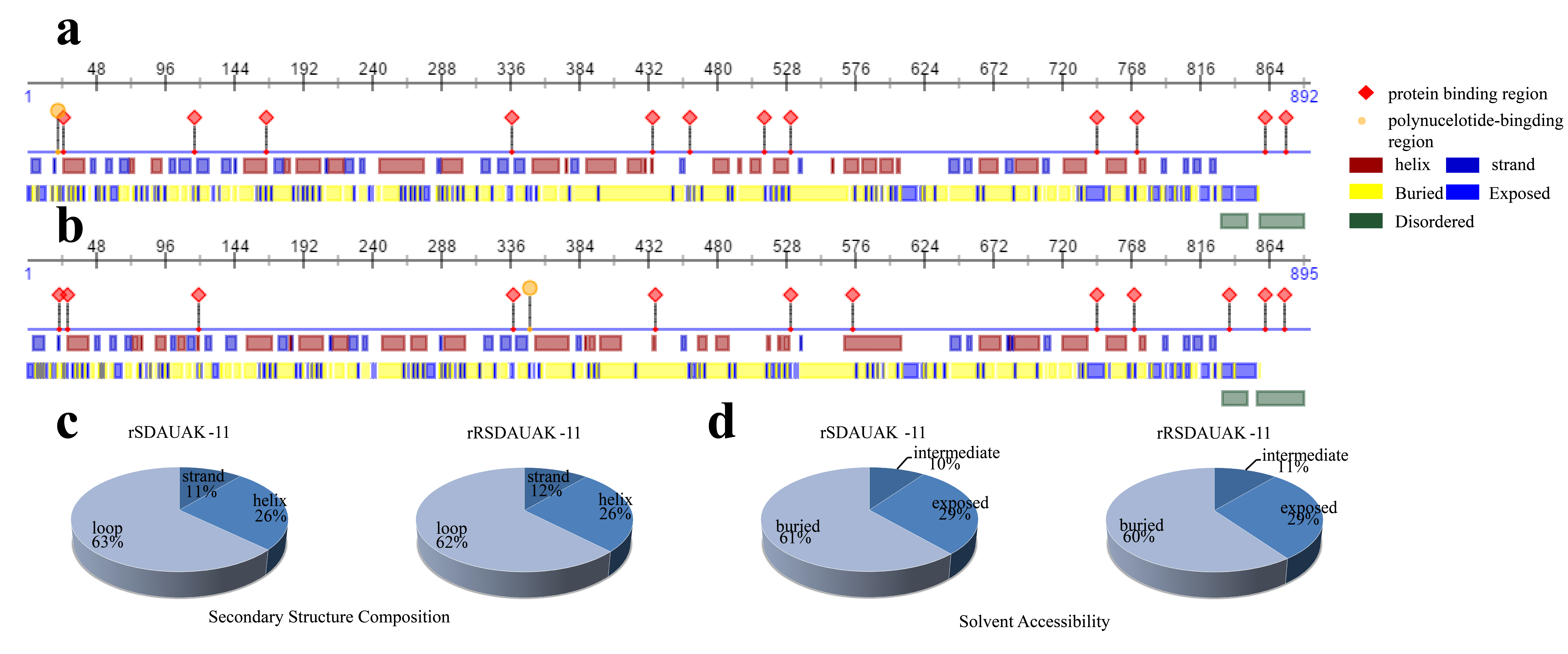

Supplement: Supplementary file 2 — Fig. S1 Secondary protein structure prediction using the online software PredictProtein (https://www.predictprotein.org/) (a) Secondary protein structure element distribution map of rSDAUAK-11 (b) sec [file 41426_2018_111_MOESM2_ESM.tif]

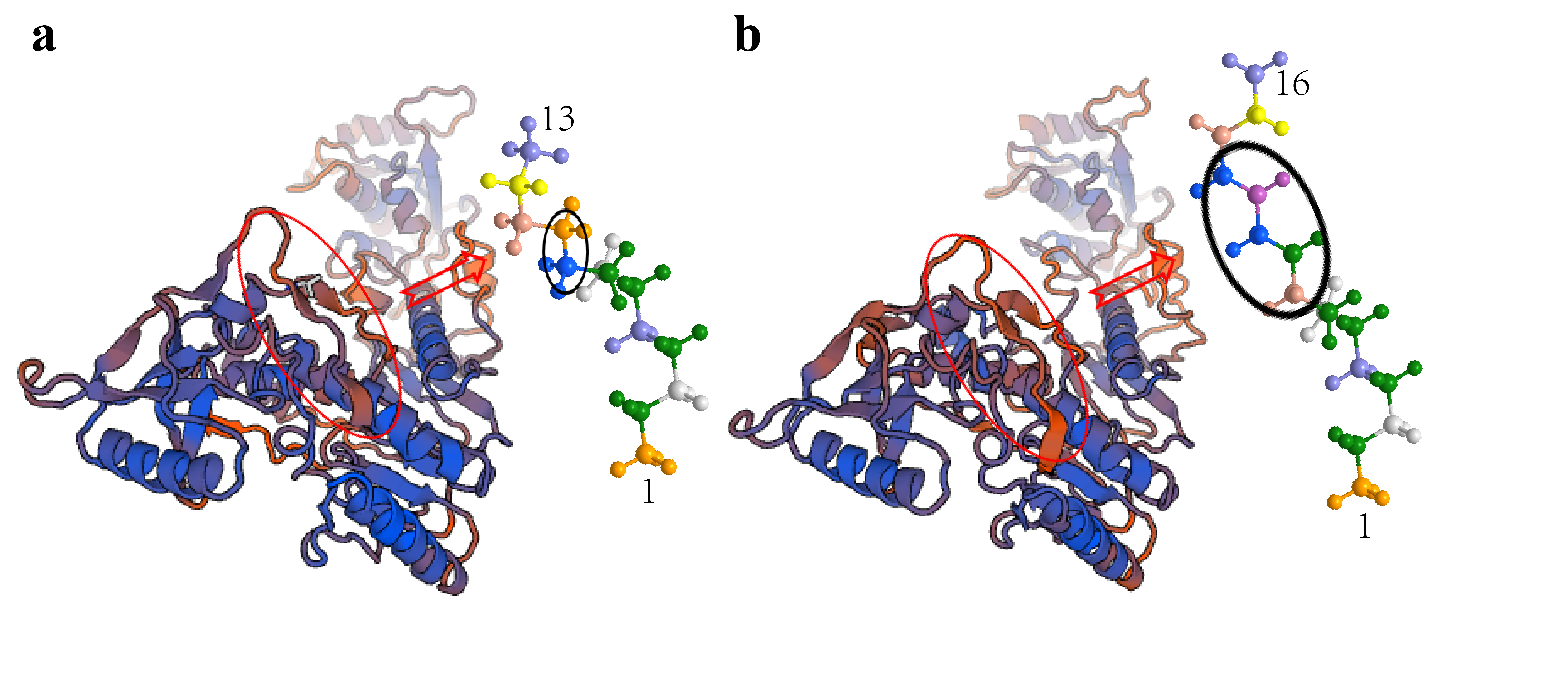

Supplement: Supplementary file 3 — Fig. S2 Tertiary protein structure prediction using the online software SWISS-MODEL (https://www.swissmodel.expasy.org/) (a) rSDAUAK-11 (b) rRSDAUAK-11; the 1-13 (rSDAUAK-11: TVALHLAIRSPDH) and 1-16 [file 41426_2018_111_MOESM3_ESM.tif]

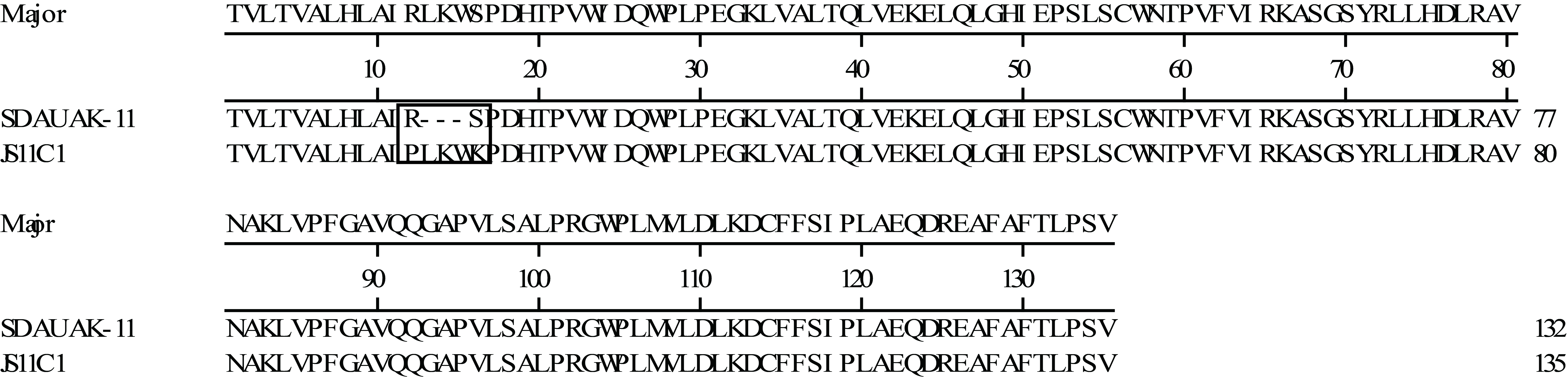

Supplement: Supplementary file 4 — Fig. S3 Amino acid sequence comparison of fragment 2714-3119 between JS11C1 and SDAUAK-11, and the difference was marked with black rectangle [file 41426_2018_111_MOESM4_ESM.tif]
